# Health Tract, No. 64

**Published:** 1862-04

**Authors:** 


HEALTH TRACT, No. 64.

### INVERTED TOE-NAIL

Is excruciatingly painful, and has repeatedly destroyed life, by mortification or lock-jaw. The nail does not grow into the flesh, but the flesh being irritated by a tight shoe, inflames and swells, crowding itself up against the sharp and unyielding edge of the nail, until it ulcerates, when the slightest touch is agonizing.

1. The old remedy was to drag out the entire nail with pincers, but even this was not always successful, terrible as it was.

2. Cut a notch in the nail down to the quick, along the center of the arch, from the root outward, or scrape it with a glass; this breaks the arch, and the pressure at the sides tends to close it up, and thus relieves, because the nail changes its curvature, and the outer edges turn up, instead of down.

3. Take equal quantities of blue vitriol (sulphate of copper) and common alum burnt; reduce them to a fine powder, mixing them together most thoroughly; then sift it through muslin; next, wash the parts well with Castile soap-suds, and apply the powder; repeat this four times every twenty-four hours.

4. Scrape the whole nail moderately with a piece of glass, so as to diminish its thickness considerably; then rub it all over well with a piece of solid nitrate of silver, moistened with a little water; then apply a hot poultice of linseed-meal, to remain until next morning, when the whole nail will be loosened, and may be removed without any pain; if not entirely loosened, make another but milder application of the caustic.

5. Scrape the toe-nail to the quick with a piece of glass, from the root outward, as near as possible to the ailing edge; then, with a pair of pincers, catch hold of the edge of the nail farthest from the sore spot, and gently draw the nail away from it toward the center, and repeat daily.

6. Freeze the parts; scrape the nail longitudinally to the quick, the eighth of an inch from the ailing edge; then with tweezers draw out the offending part; this is done without pain.

7. Spread an ointment of per-chloride of iron on some lint, and lay it over the excrescence; renew it twice daily, and in four days the excrescence becomes dry, is easily detached, and in a week all is well.

8. When there is " proud flesh " or ulceration, drop two or three drops of melted tallow between the nail and the granulations. One application usually gives immediate relief, by the hot tallow insinuating itself in every interstice under the nail, acting as a liquid cautery, the parts drying up in a few days.

9. The editor's plan is simply to insinuate, with a bodkin or silver teaspoon-handle, a small amount of lint or cotton between the edge of the nail and flesh, in the gentlest manner, and let it remain there until next day, when more is to be insinuated, and so on, until, by the absorption caused by the pressure, the swelling or proud flesh entirely disappears. If this is done when attention is first directed unpleasantly to the toe, it gets well in a day or two. If neglected until there is great pain and swelling, or ulceration, it is better to go to bed and keep the toe poulticed with bread and milk or linseed-flour, put on hot and renewed every four hours; then scrape the nail to the quick at the center, from the root outward, and proceed as above. Remember that it is best, in trimming both finger and toe-nails, not to trim down to the corners, but let the nail grow out rather more square, not rounding off at the angle. It will hasten the cure, if the cotton, after being put in, is moistened with liquid nitrate of silver, forty grains to the ounce.

